# The Shaking Palsy of the Larynx—Potential Biomarker for Multiple System Atrophy: A Pilot Study and Literature Review

**DOI:** 10.3389/fneur.2019.00241

**Published:** 2019-03-26

**Authors:** Tobias Warnecke, Annemarie Vogel, Sigrid Ahring, Doreen Gruber, Hans-Jochen Heinze, Rainer Dziewas, Georg Ebersbach, Florin Gandor

**Affiliations:** ^1^Department of Neurology, University of Münster, Münster, Germany; ^2^Hospital for Movement Disorders/Parkinson's Disease, Beelitz-Heilstätten, Germany; ^3^Department of Neurology, Otto-von-Guericke University, Magdeburg, Germany

**Keywords:** multiple system atrophy, laryngeal dysfunction, pharyngeal dysfunction, irregular arytenoid cartilage movements, FEES, dysphagia, tremulous arytenoid movements, biomarker

## Abstract

In its early stages multiple system atrophy (MSA), a neurodegenerative movement disorder, can be difficult to differentiate from idiopathic Parkinson's disease (PD), and emphasis has been put on identifying premotor symptoms to allow for its early identification. The occurrence of vegetative symptoms in addition to motor impairment, such as orthostatic hypotension and neurogenic bladder dysfunction, enable the clinical diagnosis in the advanced stages of the disease. Usually with further disease progression, laryngeal abnormalities become clinically evident and can manifest in laryngeal stridor due to impaired vocal fold motion, such as vocal fold abduction restriction, mostly referred to as vocal fold paresis, or paradoxical vocal fold adduction during inspiration. While the pathogenesis of laryngeal stridor is discussed controversially, its occurrence is clearly associated with reduced life expectancy. Before the clinical manifestation of laryngeal dysfunction however, abnormal vocal fold motion can already be seen in patients that might not yet fulfill the diagnostic criteria of MSA. In this article we summarize the current literature on pharyngolaryngeal findings in MSA and report preliminary findings from a pilot study investigating eight consecutive MSA patients. Patients showed varying speech abnormalities. Only 2/8 patients exhibited laryngeal stridor. However, during FEES, all patients presented with irregular arytenoid cartilages movements and vocal fold abduction restriction. 3/8 showed vocal fold fixation and 1/8 paradoxical vocal fold motion. All patients presented with oropharyngeal dysphagia, 5/8 with penetration or aspiration events. We suggest that specific abnormal vocal fold motion can help identifying MSA patients and may allow for delimiting this disorder from idiopathic PD. These findings therefore may serve as a novel clinical biomarker for MSA. Based on the available data and our preliminary clinical experience we developed a standardized easy-to-implement task-protocol to be performed during flexible endoscopic evaluation of swallowing (FEES) for detection of MSA-related pharyngolaryngeal movement disorders. Furthermore, we initiated a prospective study to evaluate the diagnostic utility of this protocol.

## Introduction

Multiple system atrophy (MSA) is a sporadic progressive neurodegenerative disorder characterized by Parkinsonian and cerebellar symptoms of varying severity and autonomic dysfunction. Similar to Parkinson's disease (PD), its age of onset is in the 6th decade with both sexes equally affected (1, 2). In contrast, MSA is a rapidly progressing disease, and mean survival after diagnosis is 6–10 years (3, 4). Depending on the leading presentation of the motor impairment, the disease is divided into a Parkinsonian (MSA-P) and a cerebellar sub-type (MSA-C). In a ratio of 2:1 to 4:1, MSA-P is more prevalent in the Americas, Europe and Korea (5–7). In Japan however, MSA-C is the more common (2). Currently, only symptomatic pharmacological and non-pharmacological treatments are available (1, 8).

Despite its faster progression, MSA in its early stages can be misinterpreted as PD or late-onset cerebellar ataxia and therefore poses a major diagnostic challenge (1). It is usually not until the advanced stages of the disease that autonomic failure and urogenital dysfunction become apparent (9, 10), which then, in addition to poor levodopa response, allow for the diagnosis of probable or possible MSA according to the current diagnostic criteria (11). Due to the diagnostic challenges in the early phase of the disease, emphasis has been put on identifying premotor symptoms to improve diagnostic accuracy on one hand and delineate MSA from PD or other lookalikes on the other (6, 12, 13). The European MSA study group (EMSA-SG) developed a red flag check list and analyzed the prevalence of 22 clinical features in 74 MSA and 116 PD patients (14). Of these 22, 13 showed a specifity of >95% for MSA and where grouped into six categories: early instability, rapid progression, abnormal postures (including Pisa syndrome, disproportionate antecollis and/or contractures of hand or feet), respiratory dysfunction (including diurnal or nocturnal inspiratory stridor and/or inspiratory sighs) and emotional incontinence. With two or more of six red flag categories present specificity was 98.3% and sensitivity was 84.2% in that cohort. When applying these criteria to patients with possible MSA-P, 76.5% of them would have been correctly diagnosed as probable MSA-P 15.9 ± 7.0 months earlier than with the consensus criteria alone (14). Interestingly, with 5 of these 13, nearly half of the red flag symptoms affected laryngopharyngeal functions including bulbar abnormalities such as severe dysphonia, dysarthria or dysphagia, and respiratory dysfunction including diurnal or nocturnal inspiratory stridor. This underlines the importance of assessing the laryngopharynx when suspecting MSA (15). It becomes even more significant when the clinical presentation does not yet allow for the diagnosis of MSA according to the consensus criteria. Examination of the laryngopharynx may reveal subclinical abnormalities associated with MSA and thereby allow for an earlier diagnosis. Williams stated already in 1979 that MSA patients “…should be examined routinely for laryngeal dysfunction and the examination repeated if suggestive symptoms develop….” (15). To date however, there is no standardized examination protocol for easy to implement diagnostic procedures such as flexible endoscopic laryngoscopy and examination of the swallowing (FEES) to systematically assess laryngopharyngeal symptoms in patients with suspected MSA.

This article gives an overview of the current literature on laryngeal abnormalities in MSA (summary of publications in Table 1) and pharyngeal symptoms in MSA and their potential pathophysiological mechanisms. We present a pilot study in 8 MSA patients and highlight the laryngopharyngeal findings. Based on the current data and our preliminary findings we suggest a simple structured diagnostic task-protocol to be performed during FEES for detection of MSA-related laryngopharyngeal symptoms. We furthermore suggest that irregular arytenoid cartilage movements (ACM) are specific for MSA and can serve as a clinical biomarker for the disease that may allow for differentiation of MSA from PD.

**Table 1:** Overview and frequency of typical laryngeal findings in MSA.

**Symptom**	**Frequency n/total (%)**	**References**
Stridor	34/100 (34%) MSA-P: 30/82 (37%) MSA-C: 4/18 (22%)	(18)
	36/104 (34.6%)	(17)
PVFM	1/1 (100%)	(30)
	9/10 (90%)	(29)
	1/1 (100%)	(32)
VFMI	38/38 (100%) Bilateral: 32/38 (84,2%) Unilateral: 6/38 (15.8%)	(34)
	17/36 (47.2%)	(35)
Irregular ACM	3/6 (50%)	(37)
	6/21 (28.6%)	(27)
	18/28 (64.3%)	(38)

*PVFM, paradoxical vocal fold motion; VFMI, vocal fold motion impairment; ACM, arytenoid cartilage movement*.

## Laryngeal Findings in MSA

### Inspiratory Stridor

Typically in the advanced disease stages, inspiratory stridor becomes evident (16, 17) but may manifest at any stage of the disease (18–20), with few reports of stridor being the initial (21, 22) or even the sole symptom of MSA (23, 24). Inspiratory stridor is found in up to one third of MSA patients (17–19), is therefore considered a red flag (14) and consequently was included in the updated consensus diagnostic criteria as a supportive feature (11). It can manifest as a diurnal or nocturnal symptom (14), and its occurrence is associated with reduced life expectancy (25). Furthermore, inspiratory stridor might be a predictor for sudden nocturnal death (10, 26). However, the exact underlying mechanism of sudden nocturnal death in MSA remains unresolved and considered of multifactorial origin (27).

### Paradoxical Vocal Fold Motion

**Figure 1:**
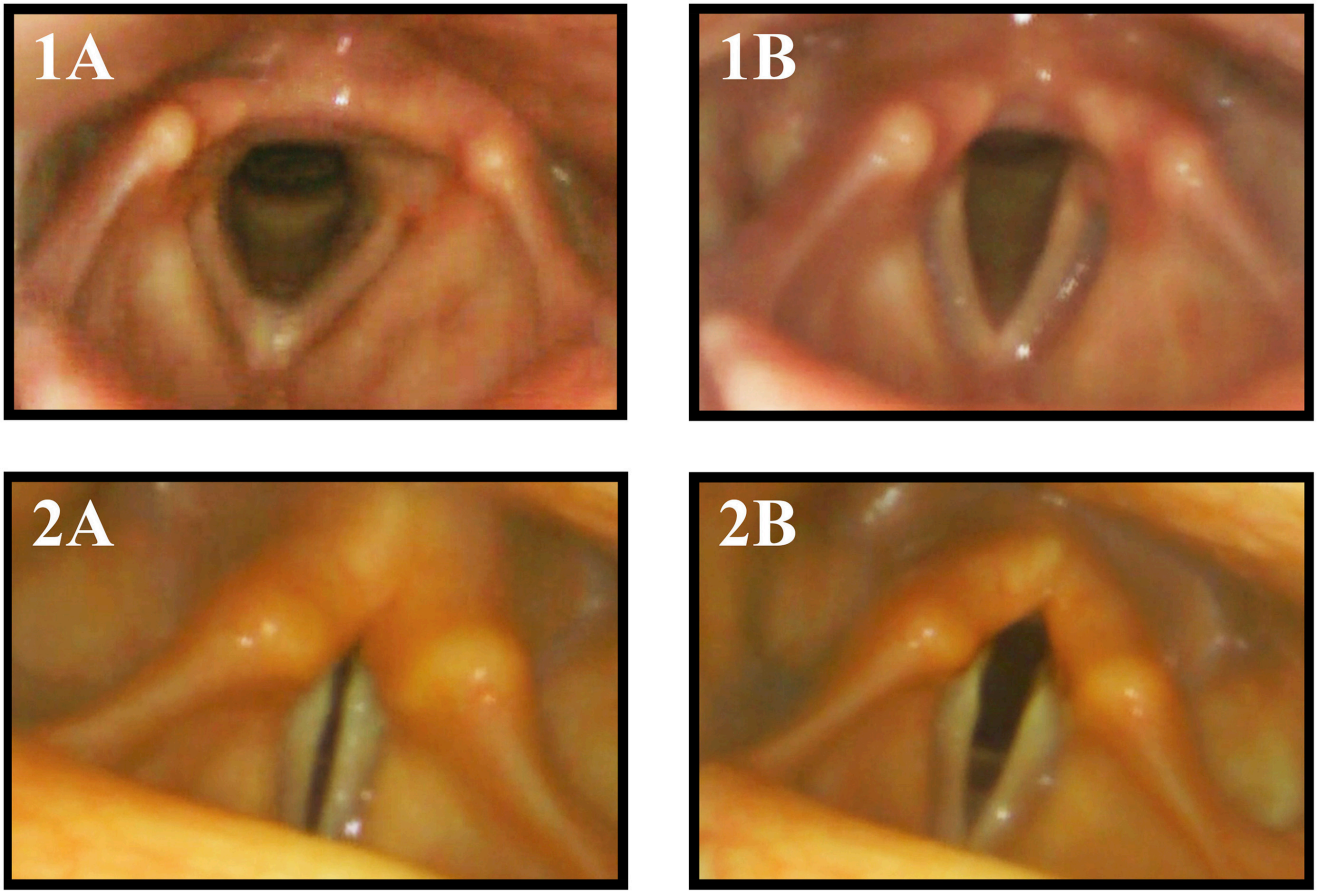
Vocal fold motion during breathing with inspiration **(A)** and expiration **(B)** in a healthy control subject (1) and MSA patient (2). The MSA patient exhibited paradoxical vocal fold motion with inspiratory stridor.

Aragane (28) first described paradoxical vocal fold motion (PVFM) during respiration in MSA patients, with adduction during inspiration and abduction during expiration (Figure 1). Isono and colleagues presented tonic thyroarytenoid muscle (TA) activation during inspiration in MSA patients (29). The TA muscle ceases activity under physiological conditions since it serves as a vocal fold adductor. Umeno described one MSA patient with normal vocal fold motion and normal EMG activity of laryngeal muscles in the wake state but paradoxical vocal fold motion during sleep with inspiratory vocal fold adduction and abduction during expiration, resulting in nocturnal stridor (30). Shiba initiated several studies presenting activation of laryngeal adductor muscles during inspiration (31–33).

### Vocal Fold Motion Impairment

**Figure 2:**
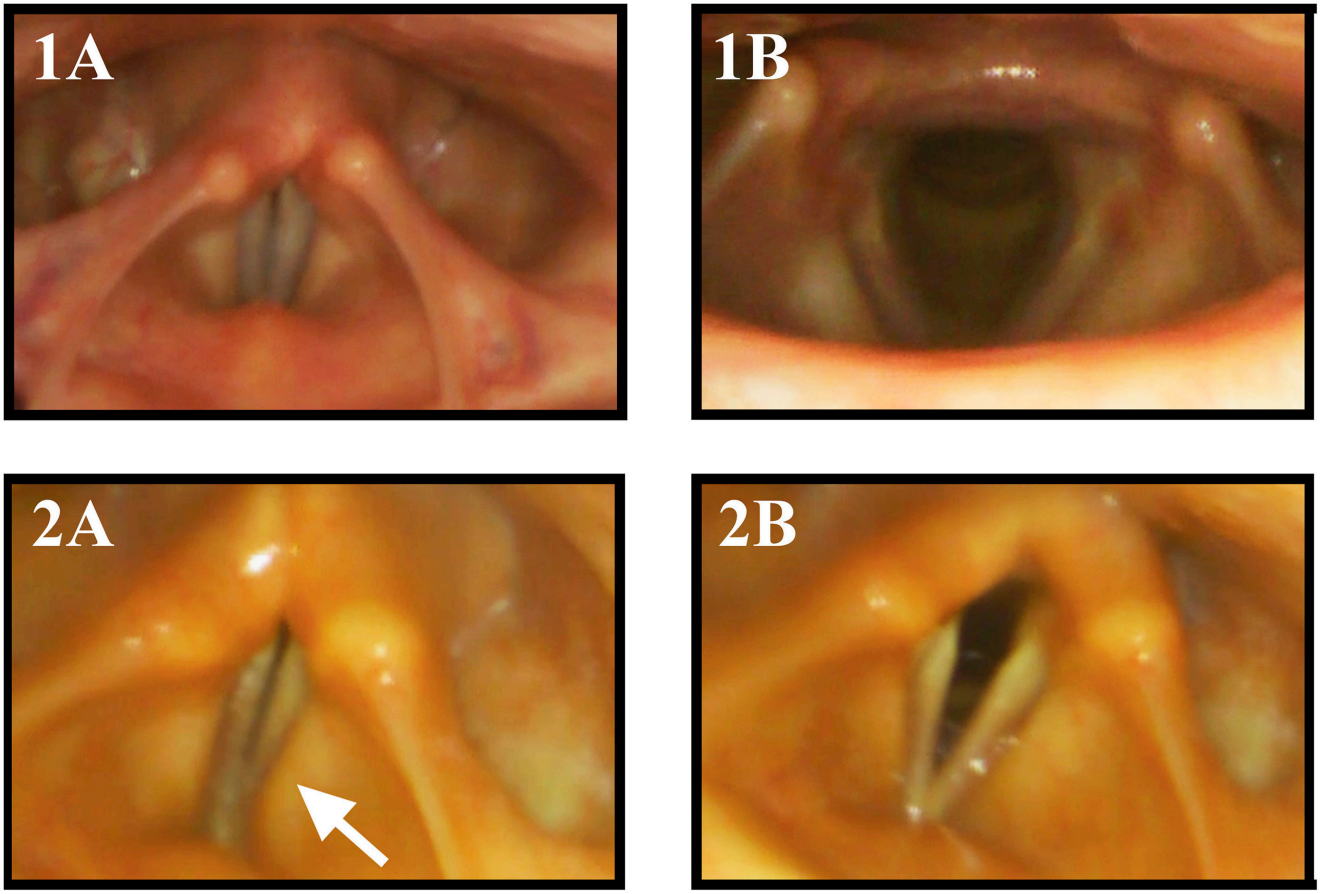
Vocal fold motion during laryngeal tasks in a healthy control subject and a MSA patient: 1 healthy control, 2 MSA patient, **(1A)** phonation “eeee,” **(1B)** sniff. The MSA patient exhibits VFMI with insufficient vocal fold adduction and activation of the vestibular folds (arrow) during phonation **(2A)** and incomplete VF abduction during sniffing maneuver **(2B)**.

A retrospective review of 38 MSA patients that underwent otolaryngologic examination revealed a bilateral vocal fold motion impairment (VFMI) in 32 patients and a unilateral VFMI in 6 patients (34). Higo and colleagues (35) performed laryngoscopy on 38 MSA patients to assess laryngeal function and found VFMI in 17 patients (Figure 2). Fourteen of these had moderate to severe bilateral vocal fold abductor restriction, two showed unilateral vocal fold fixation, one patient a bilateral vocal fold fixation in the paramedian position.

### Irregular Arytenoid Cartilage Movement

Ward was the first to describe irregular and involuntary adduction/abduction movements of the vocal folds in two MSA patients that underwent laryngoscopy and described “…quivering tonicity, pseudomyoclonic motions of the aryepiglottic folds with irregular involuntary adduction or abduction of the cords…” and “… fine quivering, tremorous motions of both arytenoids and aryepiglottic folds….” (36). Simpson and colleagues described “…flickering movements of the vocal folds…” in 3 of 6 MSA patients during laryngoscopy (37). Shimohata and colleagues observed bilateral tremulous movements of the arytenoid cartilages in 6 of 21 MSA patients during fiberoptic laryngoscopy at rest (27). Ozawa and colleagues performed fiberendoscopic laryngoscopy on 28 MSA patients and 14 healthy controls. In 18 (64.3%) MSA patients irregular tremulous movement of the arytenoid cartilages was detected, none in the healthy control group (38).

### Pathophysiological Insights Into Laryngeal Findings in MSA

Initial reports supported the hypothesis that inspiratory stridor is the clinical manifestation of vocal fold abductor paralysis (15, 39–45) in addition to the Bernoulli effect, when a sudden drop of air pressure narrows the glottic gap resulting in the characteristic acoustic phenomenon (31), similar to infantile laryngomalacia (46).

Early laryngeal EMG studies using surface electrodes supported the findings of primary denervation of the laryngeal abductor muscle. Williams observed a bilateral abduction paralysis in 8 of 12 MSA patients (15). Banister described marked atrophy of the posterior cricoarytenoid muscle (PCA) on histology in three MSA patients with significant inspiratory stridor requiring tracheostomy (43). Surface EMG recording of laryngeal muscles in five MSA patients revealed evidence for denervation of the PCA (42). Furthermore, Deguchi described two patients with reduced laryngeal abduction and attributed the finding to potential peripheral palsy of the laryngeal nerve (44). These findings were supported by one autopsy study that found loss of large myelinated fibers of the laryngeal nerve in MSA patients with abductor palsy (45). The only other autopsy study focusing on the recurrent laryngeal nerve did however not find gross abnormalities or focal changes of the recurrent laryngeal nerve (43). But why is there, next to the selective weakness of the laryngeal abductor muscle, an overactivation of adductor muscles as seen in PVFM?

An airway reflex was recently discovered that activates the adductor muscles during inspiration when inspiratory effort against a narrowed glottic gap increases (33, 47). This was shown in a study on five non-MSA-patients, four of which suffered from traumatic glottic narrowing, one from a laryngeal nerve palsy post thyroidectomy. All patients exhibited inspiratory stridor, and tonic activation of the adductor muscles was recorded during inspiration. Two patients therefore received a tracheostomy. Interestingly, when breathing through the open tracheostoma, the tonic activity of the adductor muscles during inspiration ceased but reoccurred when the tracheostoma was closed and air flow had to pass through the narrow glottic gap again (33). Similar findings were shown in one MSA patient (32). Hence, bypassing the glottic gap via tracheostomy abolishes the paradoxical activation of laryngeal adductor muscles during inspiration. In addition, several authors showed that in MSA patients exhibiting inspiratory nocturnal stridor, CPAP could abolish the tonic laryngeal adductor activation (29, 48–50). This effect might be explained by reducing the intratracheal negative pressure, thereby reducing the transglottic pressure gradient through the glottic stenosis during inspiration and the likelihood of the airway reflex to be triggered (31).

The theory of a pure peripheral nerve palsy resulting in reduced vocal fold motion is opposed by findings of simultaneous co-activation of laryngeal abductor and adductor muscles during inspiration (51, 52). The posterior cricoarytenoid (PCA) muscle, the only laryngeal abductor, is by far outnumbered by the laryngeal adductors. This dysbalance and the lack of the physiological agonistic/antagonistic play contributes to the restriction of vocal fold motion in MSA (29, 39). It is likely that co-activation of laryngeal abductor and adductor muscles results in a vocal fold abduction restriction and reduced vocal fold motion on one hand and irregular arytenoid cartilages movements (ACM) on the other.

### Treatment Options for Laryngeal Findings in MSA

#### CPAP

Several studies could show the efficacy of CPAP in abolishing laryngeal stridor in MSA patients (29, 49, 50, 53). Since CPAP is a non-invasive treatment, it should be first choice when approaching laryngeal inspiratory stridor in MSA patients. Iranzo found inspiratory pressure levels of 5–10 mbar to suffice. However, he also mentioned that only few MSA patients profit from CPAP ventilation longer than 20 weeks (49). When CPAP fails to reduce laryngeal inspiratory stridor, biPAP should be considered (48).

#### Botulinum Toxin Injection

A minimal-invasive option to treat laryngeal inspiratory stridor in MSA is botulinum toxin injection into the adductor muscle complex (TA/LCA complex). Merlo showed improvement of laryngeal stridor and of vocal fold abduction in 3 of 4 MSA patients (52). Botulinum toxin injection should however be restricted to patients without dysphagia.

#### Tracheostomy

When non-invasive measures to treat laryngeal inspiratory stridor are not able to abolish the symptom, tracheostomy should be considered (15, 25). Tracheostomy is furthermore recommended when, in addition to stridor, vocal fold motion is significantly restricted and the vocal folds fixed in a paramedian position (49). In contrast, tracheostomy could potentiate dysphagia, with aspiration necessitating the use of a cuffed cannula, resulting in cessation of speech. The additional requirement of PEG-tube feeding might significantly lower the quality of life in MSA patients (31).

#### Surgery

With vocal fold laterofixation, arytenoidectomy, and partial cordectomy, three surgical options to enlarge the glottic gap are at hand. Kenyon and Umeno reported MSA patients whose nocturnal stridor improved after laterofixation of the vocal fold (30, 54). The authors emphasized however that this method should be evaluated carefully because of the significantly increased risk of aspiration due to potential worsening of the swallowing function. In addition, the intervention itself poses risks with unexpected postoperative respiratory failure, such as laryngeal edema after cordectomy and slipping of ligature after laterofixation of the vocal fold (31). This intervention is usually performed in patients with denervation palsy of the vocal fold. In MSA patients however, it must be taken into account that the adductor muscles are still activated and exhibit force on the suture providing the laterofixation (31). Some MSA patients underwent arytenoidectomy with conflicting outcomes (30, 55).

### Laryngeal Findings in Related Movement Disorders

#### Parkinson's Disease

Vocal fold bowing is a typical cinelaryngoscopy finding in 80–93% of PD patients, which can also be identified with stroboscopic laryngoscopy (56–58). Furthermore, vocal tremor can be clinically present in about 50% of PD patients and is caused by vertical laryngeal tremor during phonatory tasks (56, 57). One case study presenting rare laryngeal findings report seven patients identified over 14 years with non-paralytic vocal fold abductor paralysis exhibiting PVFM with diurnal and nocturnal inspiratory stridor (59).

#### Spinocerebellar Ataxia

Spinocerebellar ataxia (SCA) can present with cerebellar, pyramidal, extrapyramidal and autonomic symptoms similar MSA. One study compared laryngeal findings in MSA, SCA1, and SCA3 patients and found vocal fold paralysis to be less prevalent in SCA1 and SCA3 in comparison to MSA (in 29% in SCA1 and 16% in SCA3 in contrast to 82% in MSA) (39). Furthermore, SCA patients did not present with sleep-related exacerbation of VFMI, and neurogenic atrophy occurred in all intrinsic laryngeal muscles, not solely the laryngeal abductor. In addition, none of the patients exhibited irregular ACM.

## Pharyngeal Findings in MSA

### Dysphagia in MSA

Although dysphagia is a common symptom of MSA and seems to be closely related to the disease prognosis (60), only a limited number of studies have investigated the specific characteristics of dysphagia in MSA patients in detail. It should be taken into account that most of these studies were performed in Japan, a noteworthy fact when considering the higher prevalence of MSA-C in comparison to MSA-P (2). Similar to laryngeal abnormalities, swallowing is often impaired early in the disease (61, 62). There are varying reports about time of onset of dysphagia during the course of MSA. Isono and colleagues reported dysphagia in MSA-C patients to begin 4.6 ± 3.5 years after disease onset (61). Petrovic in contrast described a much later onset of dysphagia in MSA-P patients at 9.0 ± 3.7 years (63). A recent study in 59 Korean MSA patients showed a much earlier dysphagia onset in both MSA sub-types (MSA-P: 2.94 ± 1.43 years, MSA-C: 3.05 ±1.24 years) (64).

Since the act of swallowing consists of four phases (65), one might raise the question whether a certain phase is typically impaired in MSA. Taniguchi investigated 13 MSA-C and 3 MSA-P patients. All patients showed an impaired esophageal phase presenting with intraesophageal food stagnation, which potentially entails aspiration leading to pneumonia or even bolus regurgitation with bolus aspiration (66). Higo performed videofluoroscopy on 22 MSA-C and 7 MSA-P patients assessing the swallowing performance (67). The evaluation revealed a disturbed oral phase with delayed bolus transport in 73%, reduced tongue base movement in 55% and impaired oral bolus control in 49% of patients. Abnormalities in the pharyngeal phase however were detected in only 20% of the patients. 25% of these patients had a history of aspiration pneumonia. Interestingly, disease severity rather than age or disease duration were identified as risk factors for aspiration pneumonia (67). These results are in line with the findings by Isono who showed that pharyngeal dysphagia severity did not correlate with disease duration (61). An analysis of videofluoroscopic examinations in 59 MSA patients showed pharyngeal symptoms to be more often disturbed than oral symptoms. 89.8% of the patients presented with vallecular residue. Penetration or aspiration occurred in 67.8%, delayed pharyngeal swallow was observed in 57.6%. In addition, pharyngeal apraxia and vallecular residue were observed more frequently in the MSA-P sub-type and were more severe in comparison to MSA-C patients. Frequency of oral phase symptoms did not differ between MSA sub-types, with inadequate mastication in 32% and premature spillage in 23.7%.

### Treatment of Dysphagia in MSA

To date there are no treatment guidelines for MSA-related dysphagia. Furthermore, there are very few clinical studies addressing treatment strategies of dysphagia in MSA.

Firstly, levodopa response of dysphagia should be evaluated, despite the fact that levodopa responsiveness on motor symptoms is usually considered poor in MSA. However, up to 31.2% of MSA patients show a beneficial levodopa response of symptoms for a mean duration of 3.5 years (3) and might even exhibit motor fluctuations (68–70). Since adjusting dopaminergic therapy is the least invasive treatment option, a structured FEES-levodopa test should be performed to evaluate swallowing in the ON- and OFF-state (71, 72). When levodopa responsiveness has been ruled out, treatment of mild or moderate dysphagia should encompass postural maneuvers to facilitate swallowing, such as the chin tuck maneuver (71). Moreover, behavioral changes with slowing of eating, reduction of meal volumes or changing food consistencies should be considered (73). When intraesophageal bolus transport is disturbed and overactivation of the distal esophagus sphincter suspected, botulinum toxin injection into the distal esophagus has proven to alleviate dysphagia severity in PD patients (74). With the results of Taniguchi who reported intraesophageal food stagnation (66), botulinum toxin injection could be considered. When severe dysphagia with repetitive aspiration and aspiration pneumonia is present, avoidance of the oral route with a PEG-tube should be discussed with the patient to guarantee adequate nutrition and hydration (75).

## Pilot Study

### Standardized Task Protocol for Pharyngolaryngeal Abnormalities in MSA

To date, no structured protocol has been suggested to evaluate MSA specific findings in the laryngopharynx (35). Especially in diseases that pose diagnostic challenges, structured examination procedures are necessary (71). We developed a simple FEES-MSA-protocol that allows for assessing laryngeal functions and oropharyngeal performance (Table 2).

**Table 2:** Tasks of the FEES-MSA-protocol, assessed functions, and possible findings.

	**Task**	**Physiological function**	**Possible findings**
Laryngeal tasks	Normal breathing	Inspiration:mild VF abduction Expiration:VF relaxation with Mild VF adduction	VFMI Uni- or bilaterally reduced VF movement during inspiration and/or expiration with maximal amplitude to median, paramedian or intermediate position VF fixation Uni- or bilateral lack of respiration-linked VF motion with fixed position in median, paramedian, intermediate, prelateral or lateral PVFM Inspiratory VF adduction with glottic narrowing Irregular ACM Uni- or bilateral irregular movements of the arytenoid cartilages
	Fast and deep inhale	VF abduction	VFMI PVFM Irregular ACM pre and post maneuver
	Sniff through nose	VF abduction	VFMI PVFM Irregular ACM pre and post maneuver
	Phonation of “eee”	VF adduction	VFMI irregular ACM pre and post maneuver
	Imagined non-voiced “eee” (“Prepair youself or imagine to say “eee” without really saying it.”)	VF adduction	Irregular ACM pre, during and post maneuver
	sniff - “eee”- sniff – “eee”	VF adduction/ abduction and VF diadochokinesis	VFMI PVFM Irregular ACM pre, during and post maneuver
Swallowing tasks	Dry swallow	Clearing of secretion	Pharyngeal residue Penetration/aspiration
	Oral bolus control (“Keep the water in your mouth until I say to swallow.”)	Bolus control without spillage	Premature spillage
	Swallowing of (1) Pudding (2) Liquid (3) Solid consistencies (4) Placebo tablet	Swallowing function for different consistencies	Piecemeal deglutition Premature spillage Pharyngeal residue Penetration/aspiration
	Therapeutic maneuver e.g., Chin tuck maneuver (“Swallow the bolus keeping your neck upright and your chin down.”)	Potential improvement of impaired swallowing	Improvement of/unchanged swallowing function

*VFMI, vocal fold motion impairment; VF, vocal fold; PVFM, paradoxical vocal fold motion; ACM arytenoid cartilage movement*.

### Methods

This study was approved by the local Ethics Committee of the Brandenburg Medical Board (S21(a)/2017) and is conducted in accordance with the Declaration of Helsinki (76). After written informed consent was obtained, eight consecutive patients with possible or probable MSA according to the Gilman criteria (11) underwent examination. Speech characteristics were assessed by auditive analysis. All patients underwent flexible endoscopic evaluation of the swallowing (FEES). FEES equipment consisted of a 3.9-mm-diameter flexible fiberoptic rhinolaryngoscope (Olympus ENF-VH), a video processor (Olympus CV-170), and processing software (rpSzene 10 on Panel-PC-226/227) or a 2.9 mm-diameter flexible fiberoptic rhinolaryngoscope (Storz CMOS) with a portable video processor (Storz CMAC) linked to a 19″ flat screen monitor (Storz 9519NB). FEES was performed as previously described (72).

### Specific FEES-MSA-Protocol

To assess MSA specific impairment of the laryngopharynx, patients underwent the FEES-MSA-protocol allowing for a standardized endoscopic evaluation of the pharyngolaryngeal and swallowing function. The protocol is divided into an examination of the laryngeal function at rest and in action, followed by an evaluation of the swallowing.

#### Evaluation of the Larynx at Rest

After insertion of the rhinolaryngoscope, the tip of the endoscope is placed into the mid pharyngeal cavity to allow for full inspection of laryngeal structures. Firstly, the larynx is examined at rest during normal breathing for at least 2 min. Mobility of the vocal folds and arytenoid cartilages in relation to the breathing cycle is recorded. Notion is taken of any uni- or bilateral abnormal desynchronized movement. Vocal fold motion impairment (VFMI) is noted either in the inspiratory, expiratory or both phases. The position of the vocal folds and degree of maximum opening (median, paramedian, intermediate, prelateral, lateral position) is recorded. Paradoxical vocal fold motion (PVFM) with e.g., vocal fold adduction during inspiration, and irregular ACM at any time of the examination are recorded. Vocal fold fixation and its configuration is captured.

#### Evaluation of the Larynx in Action

To examine the degree of glottic opening, the patient is asked to repeatedly 1) inhale deeply and 2) sniff thereby performing vocal fold abduction tasks. To evaluate glottic closure, the patient is asked to phonate a prolonged “eee”, thereby performing a vocal fold adduction task. To examine the maximum amplitude of the vocal fold motion, the patient is then asked to perform these two tasks alternately. Furthermore, these tasks can initiate or aggravate irregular ACM.

Next, the patient is asked to perform a postural action of the vocal folds by imagining vocalizing an “eeee”. In our experience this postural task can initiate or aggravate irregular ACM.

#### Evaluation of the Swallowing

The third part comprises a supplemented standardized FEES protocol performing one dry swallow, 11 consecutive standardized test boli and a minimum of one swallow performing an individual therapeutic maneuver. Consistencies are applied in the following order: (1) three teaspoonful of green jelly (appr. 8 mls each), (2) one teaspoonful of blue-dyed liquid (appr. 5 mls) to test for oral bolus control, (3) three teaspoonful of blue-dyed liquid, (4) one sip of blue-dyed liquid from a glass, (5) three pieces of bread with butter (about 1 inch square), (6) one swallow of a placebo tablet ingested with either blue-dyed liquid or green jelly. Depending on the individual findings, a specific therapeutic swallow maneuver is performed at the end.

Swallowing function was evaluated as previously described (Table 3) (71) with the addition of documentation of fragmented swallows, i.e., piecemeal deglutition (more than one swallow needed to clear bolus from the oral cavity). To score dysphagia severity we applied the endoscopic severity of dysphagia scale, utilized in Parkinson's disease (Table 4) (72).

**Table 3:** Scores of swallowing function parameters (71).

**Score**	**Premature spillage**	**Pharyngeal residue**	**Penetration/aspiration**
0	Bolus behind tongue	No residue	No penetration/aspiration event
1	Bolus at the base of tongue or valleculae	Coating, no pooling	Penetration with protective reflex
2	Bolus moves to lateral channels or tip of epiglottis	Mild pooling, fills less than half of the cavities	Penetration without protective reflex
3	Bolus is in the piriform sinus or touches laryngeal rim	Moderate pooling, fills the cavities	Aspiration with protective reflex
4	Bolus falls into laryngeal vestibule	Severe pooling, overflows the cavities	Aspiration without protective reflex

**Table 4:** Endoscopic severity of dysphagia scale (72).

**Score**	**Classification of dysphagia severity**
0	No relevant dysphagia
1	Mild dysphagia (premature spillage and/or residues, but no penetration/aspiration events)
2	Moderate dysphagia (penetration/aspiration events with one consistency)
3	Severe dysphagia (penetration/aspiration events with two or more consistencies)

### Results

Four female and 4 male MSA patients underwent the FEES-MSA-protocol. Duration of performing the FEES-MSA-protocol varied from 15-20 min.

Seven patients suffered from MSA-P, one patient from MSA-C. Mean age was 59.9 ± 6.9 years, mean disease duration 3.0 ± 1.2 years, mean Hoehn and Yahr stage 3.75 ± 1.04 (Table 5). Patients exhibited a broad variety of speech pathology. Four (50%) patients showed fluctuating pitch when phonating, only one patient presented with high-pitched voice. 5/8 patients showed hypokinetic-rigid dysarthria of varying severity. The patient presenting with MSA-C showed an ataxic type of dysarthria. One patient exhibited a mixed type of dysarthria (Table 5). Only two (25%) patients exhibited diurnal inspiratory laryngeal stridor as a clinical sign of laryngeal pathology.

**Table 5:** Patients' demographic data and speech characteristics.

**Patients' characteristics**					**Speech characteristics**			
**#**	**Age, sex**	**Disease duration in years**	**MSA sub-type**	**Hoehn-Yahr stage**	**Hypophonia**	**Pitch**	**Diurnal stridor**	**Dysarthria**
1	51, f	4	MSA-P	4	+	Fluctuating	−	Severe Hypokinetic-rigid
2	61, f	4	MSA-P	2	+	Fluctuating	+	Normal
								
3	58, f	3	MSA-P	5	+	Normal	−	Severe Mixed
4	63, f	2	MSA-P	5	+	Fluctuating	−	Moderate Hypokinetic-rigid
5	54, m	4	MSA-P	4	+	Fluctuating	+	Moderate Hypokinetic-rigid
6	57, m	1	MSA-C	3	+	High	−	Severe Ataxic
7	74, m	2	MSA-P	4	+	Normal	−	Moderate hypokinetic-rigid
8	61, m	4	MSA-P	3	+	Normal	−	Severe Hypokinetic-rigid

However, during flexible endoscopic evaluation of the laryngeal and swallowing function, all patients showed irregular ACM. In 6 (75%) patients, irregular ACM was present at rest (Supplementary Video 1). The remaining 2 (25%) showed irregular ACM during provoking maneuvers (sniff, deep inhale, phonation “eee” and imagined non-voiced “eee”) (Supplementary Video 2). In 2 (25%) patients with irregular ACM at rest, the movements were enhanced during provoking maneuvers (Supplementary Video 2). Furthermore, all patients exhibited vocal fold abduction restriction (Figure 2). Three (37.5%) patients showed a vocal fold fixation in the paramedian position and one patient presented paradoxical vocal fold motion (Figure 1).

When evaluating the swallowing, 5 (62.5%) patients presented with piecemeal deglutition for all consistencies. Seven (87.5%) patients showed premature spillage with 6 events for pudding, 5 for liquids and 2 for solids. All patients showed pharyngeal phase dysfunction. Six patients showed pharyngeal residues in the valleculae with four events for solids, 3 for pudding and 1 for liquids. Five (62.5%) patients showed penetration/ aspiration events. The first of these patients presented with penetration/aspiration score 1 for pudding and score 3 for liquids, the second showed score 1 for pudding and liquids, the third showed score 1 for all consistencies, the fourth presented with score 3 for liquids and the fifth showed score 2 for liquids. Characteristics of laryngeal findings and swallowing function did not differ between the MSA sub-types (Table 6).

**Table 6:** Endoscopic pharyngolaryngeal findings performing the MSA-FEES-protocol in 8 MSA patients.

	**Flexible endoscopic evaluation of the larynx**						**Flexible endoscopic evaluation of swallowing (FEES)**		
**#**	**VF abduction restriction**	**VF fixation**	**PVFM**	**Irregular ACM**	**Irregular ACM provoked**	**Premature spillage score**	**Pharyngeal residue score**	**Penetration/aspiration score**	**Dysphagia severity score**
1	+	–	+	+	–	3	0	0	1
2	+	–	–	–	+	3	2	1	2
3	+	–	–	+	–	4	2	3	2
4	+	–	–	+	–	3	0	3	2
5	+	+	–	–	+	0	0	0	1
6	+	+	–	+	–	4	0	1	2
7	+	–	–	+	+	2	2	2	3
8	+	+	–	+	+	2	2	0	1

*VF, vocal fold; PVFM, paradoxical focal fold motion; ACM, arytenoid cartilage movements*.

## Discussion

The structured FEES-MSA-protocol was well tolerated by all patients. With a duration of about 20 min, the examination was easy to implement into the diagnostic routine.

Patients presented with MSA typical speech characteristics of varying severity (77). However, in our cohort, all MSA patients exhibited laryngeal and pharyngeal abnormalities on flexible endoscopic evaluation, regardless of the severity of clinical speech pathology. More so, the severity of clinical speech impairment did not correlate with the laryngeal abnormalities detected while performing the FEES-MSA-protocol. Irregular ACM at rest were present in 75% of patients. These findings are in line with previous results from laryngoscopy studies that found irregular ACM in 50–100% of cases (27, 36–38). Irregular involuntary movements have been described in MSA patients to occur predominantly in hands and fingers. Some authors stated that these involuntary jerky movements are underrecognized in MSA patients (78–80). Similar to these irregular jerky and tremulous movements of the limbs, these movements might not be present at rest but can be observed during postural action. Salazar investigated 11 patients with MSA. Only one patient showed Parkinsonian pill-rolling tremor of the hand at rest. However, during postural maneuvers, irregular jerky movements of the hands were present in 9 (82%) patients (80). With this clinical observation in mind we designed a “postural” task for the larynx and could show that in those two patients without irregular ACM at rest, ACM could be provoked during this “postural” maneuver.

In addition, all patients showed vocal fold abduction restriction. Despite this laryngoscopic finding, only two patients demonstrated diurnal inspiratory stridor on clinical examination. This finding of vocal fold motion impairment resulting in a narrow glottic gap is in line with previous results reported by Lalich, who investigated 38 MSA patients and found 32 to present with a bilateral VFMI, the remaining 6 with a unilateral VFMI (34).

Since the prevalence of irregular ACM in our MSA cohort was 100% we do believe that ACM might serve as clinical biomarker for MSA, potentially allowing for differentiating MSA from other disorders. This finding is even more relevant when bearing the young mean age and short disease duration of our cohort in mind. Irregular ACM was found as early as 1 year after disease onset and was present in both MSA-P and MSA-C subtypes. In contrast to PVFM or inspiratory stridor (59), irregular ACM have not been described in other movement disorders. There are numerous publications on tremor affecting the larynx [for review see: (81–83)]. However, these tremor forms show rhythmic and regular movements of laryngeal structures as opposed to the findings we observed in this pilot study.

Dysphagia was present in all patients, regardless of disease duration or MSA sub-type. These findings are in line with previous results (61, 66, 67). Although not useful as a specific biomarker, dysphagia however remains a clinically relevant symptom associated with survival in MSA patients (60, 63). Since dysphagia is preclinically detectable by instrumental assessment tools (67), an early diagnosis is vital for preventing dysphagia-related complications.

We here present results from a pilot study. Despite the small sample size and lack of a control group, we propose an interesting hypothesis. We are currently conducting a trial in a larger cohort of MSA patients and compare findings to PD patients to further support the theory that irregular ACM might serve as a clinical biomarker allowing for differentiating MSA from PD.

## Conclusion

Pharyngolaryngeal abnormalities have a high prevalence in MSA. Our findings suggest that characteristic pathologic findings can be revealed on flexible endoscopy before becoming evident clinically when implementing the FEES-MSA-protocol. Irregular arytenoid cartilages movement (ACM) at rest or with provoking maneuvers might serve as a diagnostic clinical biomarker and facilitate identification of MSA patients. Since FEES is now widely established in many Neurology departments we strongly suggest that all patients with suspected MSA should undergo this procedure to look for disease specific findings that support the diagnosis and enable adequate treatment as early as possible (84). Utilizing a structured FEES-MSA-protocol will help assessing relevant functions and identify MSA-specific abnormalities such as irregular ACM, PVFM, VFMI, premature spillage, or pharyngeal residue. A prospective study is under way to evaluate the diagnostic utility of this protocol.

## Data Availability

All datasets generated for this study are included in the manuscript and/or the Supplementary Files.

## Author Contributions

FG: ethics application. FG, AV, and TW: conception and design, acquisition, analysis, and interpretation of data, retrieving review data, manuscript writing, and editing. SA: acquisition, analysis and interpretation of data. DG: acquisition, analysis, and interpretation of data, editing manuscript. H-JH, RD, and GE: conception and design, review data, editing manuscript.

### Conflict of Interest Statement

The authors declare that the research was conducted in the absence of any commercial or financial relationships that could be construed as a potential conflict of interest.

## Supplementary Material

Supplementary Video 1 Irregular arytenoid cartilages movements at rest in MSA patient.

Supplementary Video 2 Irregular arytenoid cartilages movements during postural maneuver in MSA patient.
